# Supplementary figures and images for: Multicenter international assessment of a SARS-CoV-2 RT-LAMP test for point of care clinical application
Source: PLoS One. 2022 May 11;17(5):e0268340. doi: 10.1371/journal.pone.0268340 (PMC9094544; doi:10.1371/journal.pone.0268340)

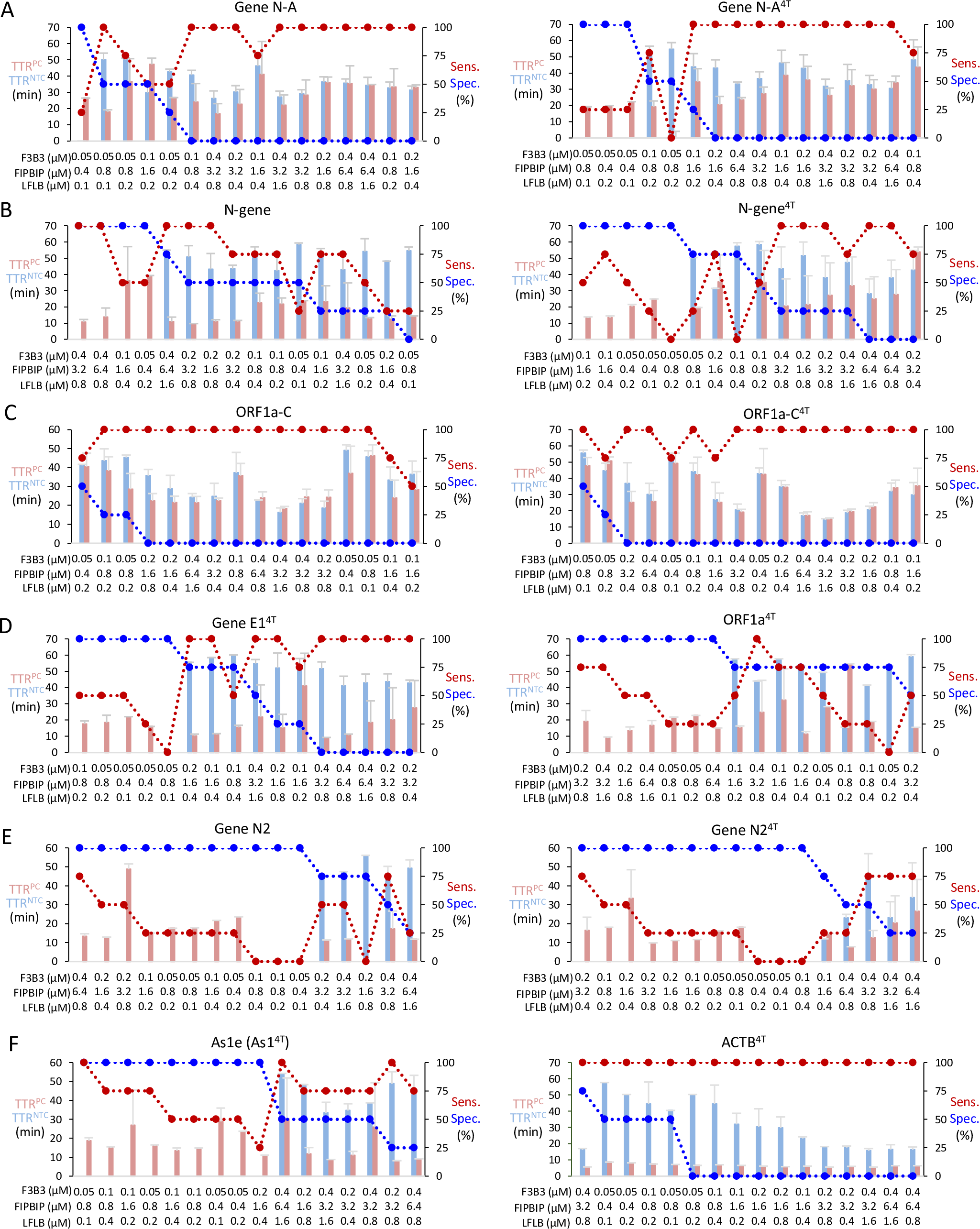

Supplement: S1 Fig — Primer sets for: (A) GeneN-A and GeneN-A4T; (B) N-gene and N-gene4T; (C) ORF1a-C and ORF1a-C4T; (D) Gene E14T and ORF1a4T; (E) Gene N2 and Gene N24T; (F) As1e and ACTB4T. NTC, no template control; PC, positive control (30 copies of SARS-CoV-2 RNA); TTR, time to results (min); Error bars represent mean ± standard deviations. (TIF) [file pone.0268340.s001.tif]

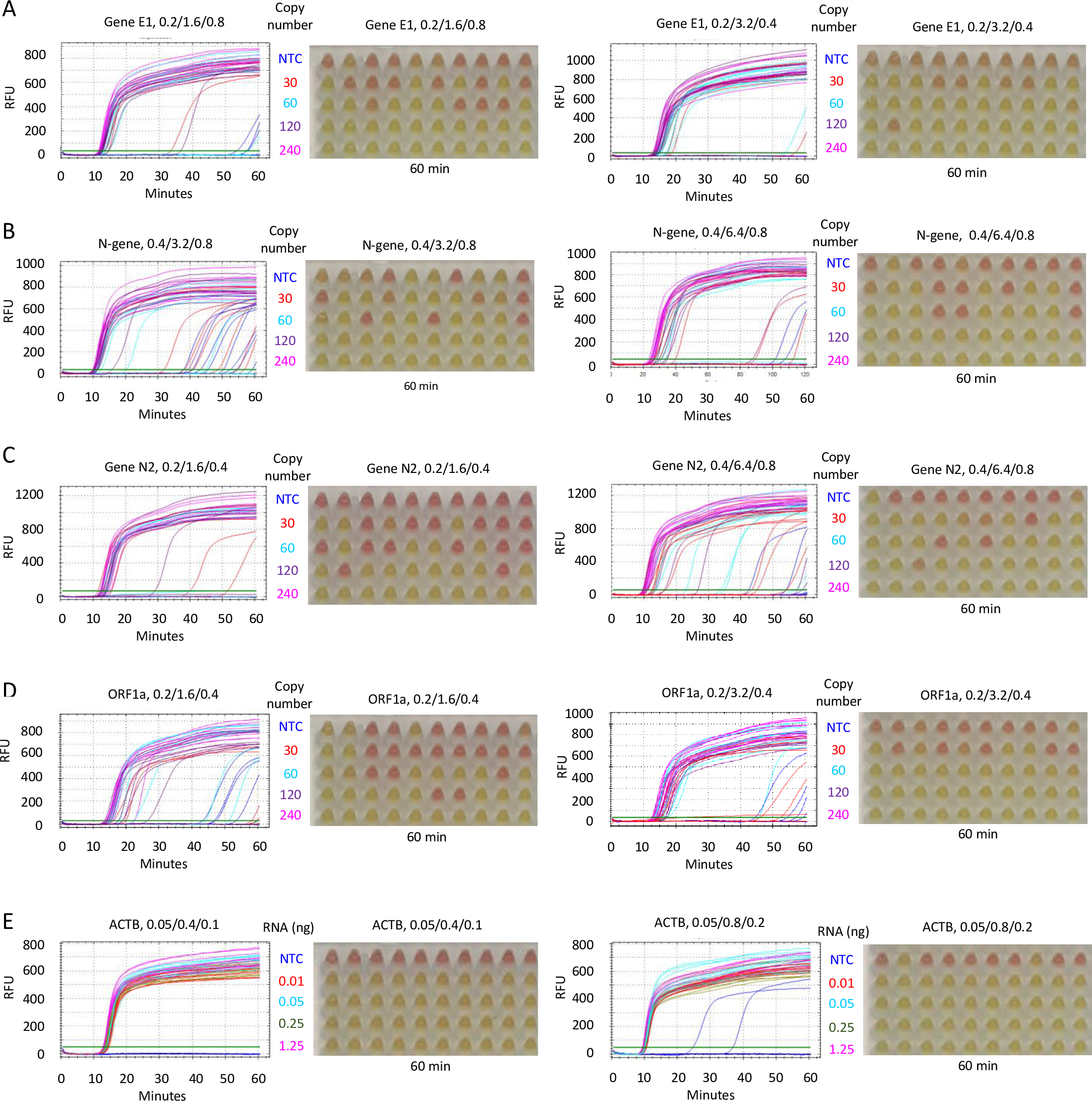

Supplement: S2 Fig — Fluorescent readouts and images of color changes at 60 minutes are shown for the following primer sets: (A) Gene E1. (B) N-gene. (C) Gene N2. (D) ORF1a. (E) ACTB. Primer amounts (F3B3/FIPBIP/LFBF, μM) are indicated above each assay. Each condition was evaluated with 10 replicates. RFU, relative fluorescence units; NTC, no template control. (TIF) [file pone.0268340.s002.tif]

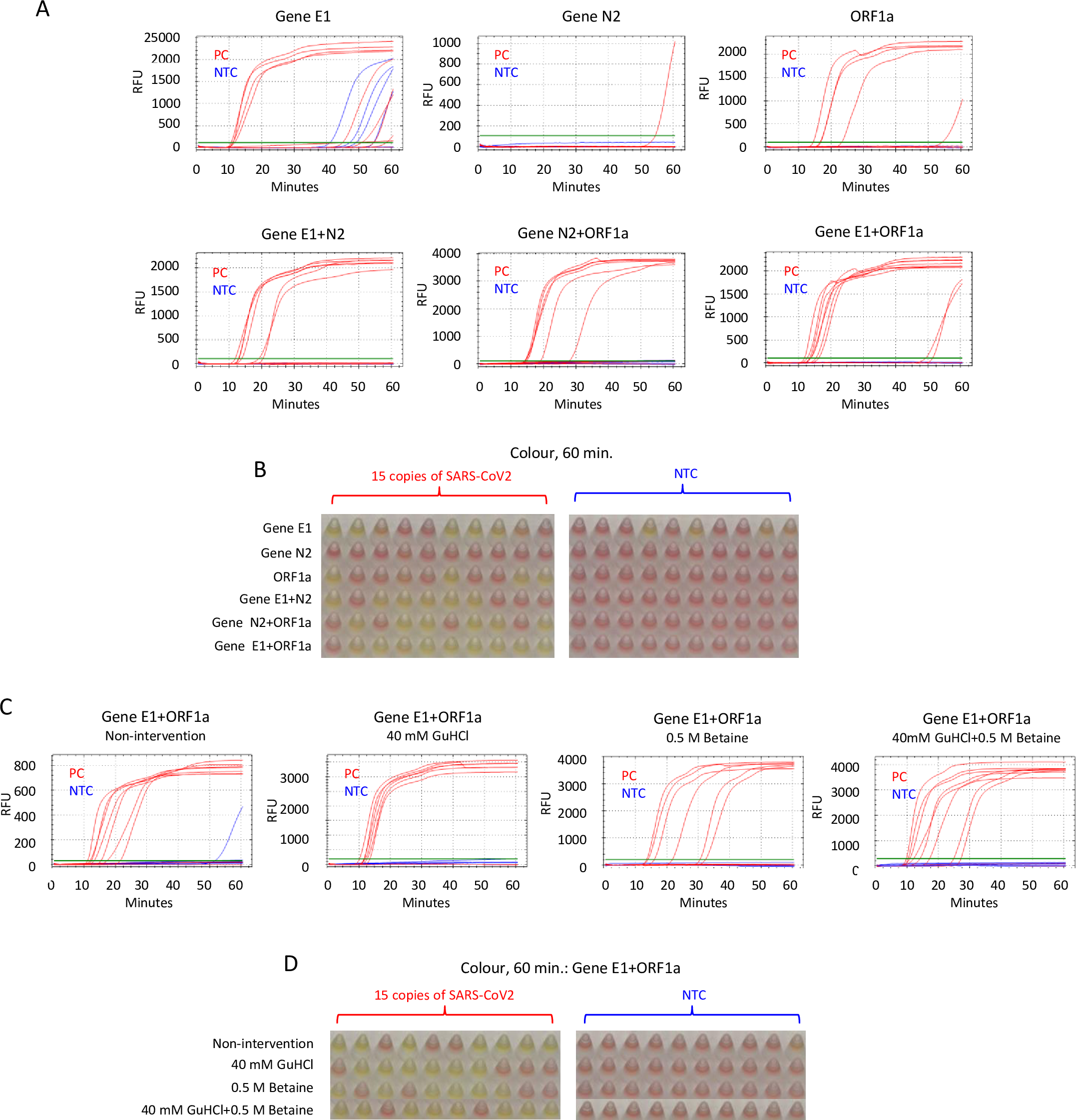

Supplement: S3 Fig — (A) Fluorescent readouts of RT-LAMP with the indicated primer multiplexing (also see Fig 3A). (B) Phenol red colour at 60 minutes from assays in (A). (C) Fluorescent readouts of RT-LAMP with multiplexed primer sets for Gene E1 and ORF1a with the indicated supplements (also see Fig 3B). (D) Phenol red colour at 60 minutes from assays in (C). RT-LAMP reactions were performed with 15 copies of SARS-Cov-2 RNA, and each condition was evaluated with 10 replicates. NTC, no template control; RFU, relative fluorescence units. (TIF) [file pone.0268340.s003.tif]

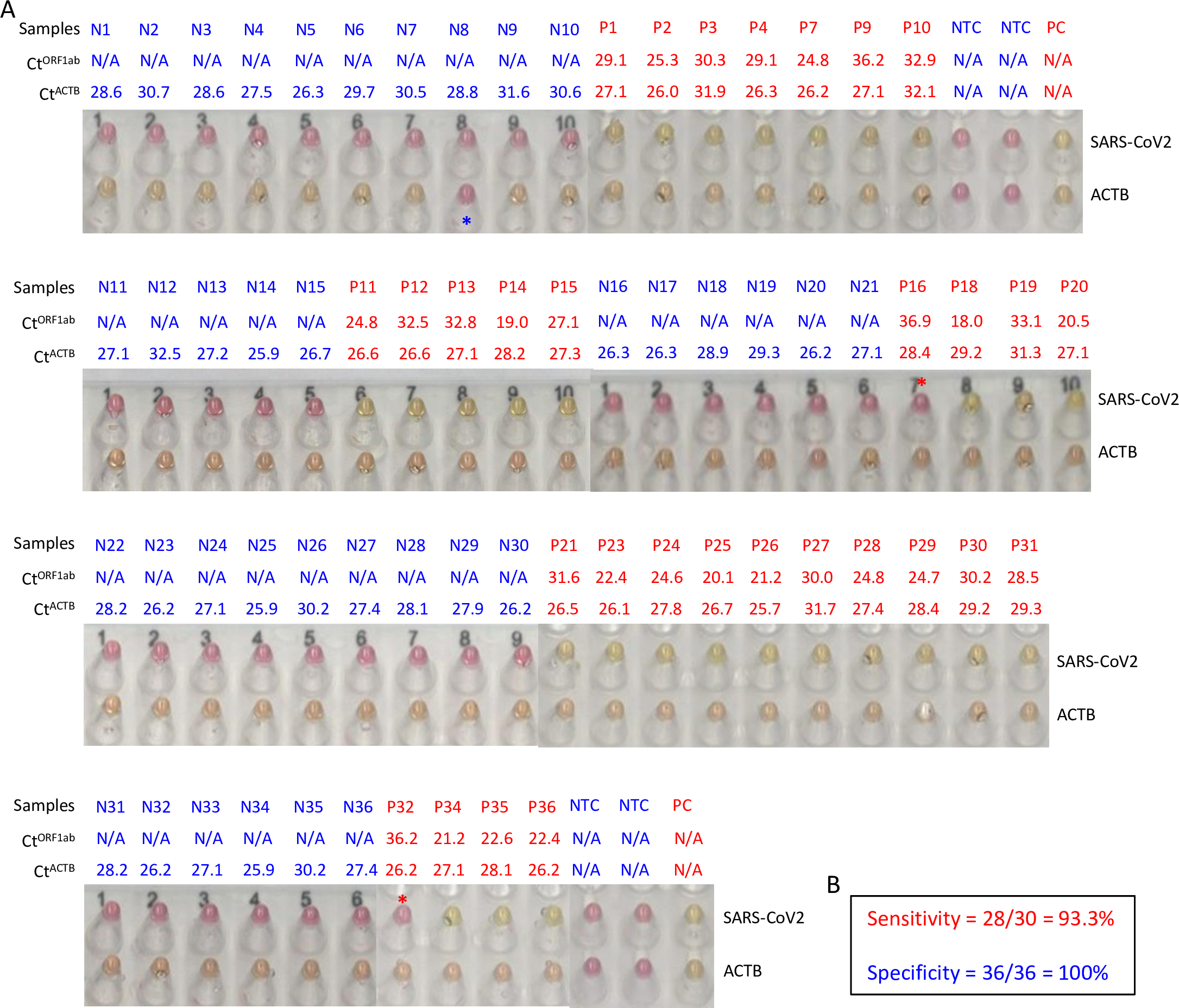

Supplement: S4 Fig — (A) Phenol red colour of RT-LAMP assays with extracted RNA from clinical NP samples. RT-LAMP was carried out in a water batch at 65°C for 25 minutes with multiplexed Gene E1 and ORF1a primers and 40mM and 0.5M betaine. (B) Sensitivity and specificity in (A). NTC, no template control; PC, positive control (240 copies of SARS-CoV-2 and 1ng human RNA). (TIF) [file pone.0268340.s004.tif]

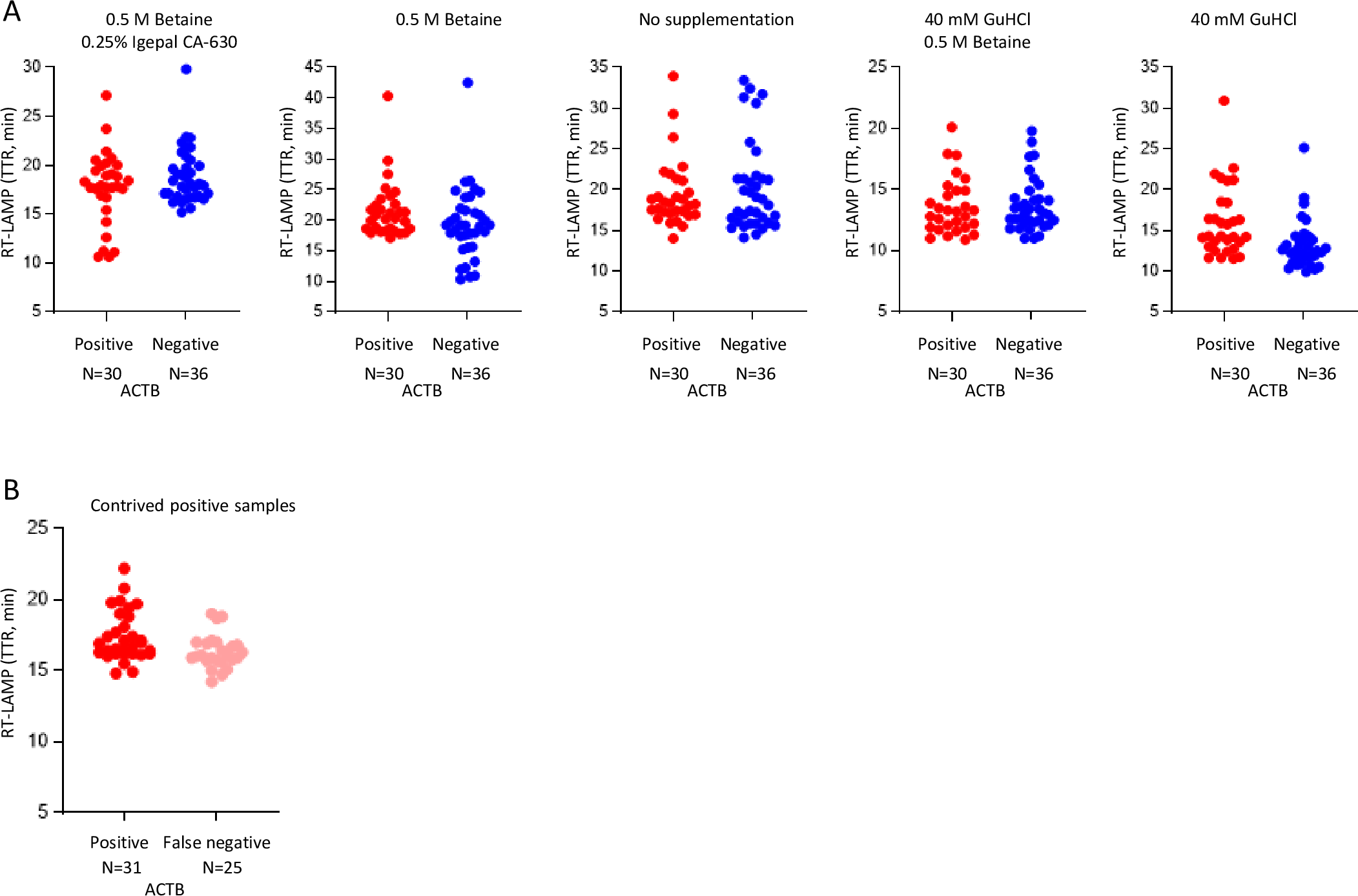

Supplement: S5 Fig — (A) Distribution of ACTB TTRs of raw clinical NP samples under the indicated RT-LAMP conditions. (B) Distribution of ACTB TTRs between RT-LAMP test positive and negative from contrived raw positive NP samples. (TIF) [file pone.0268340.s005.tif]

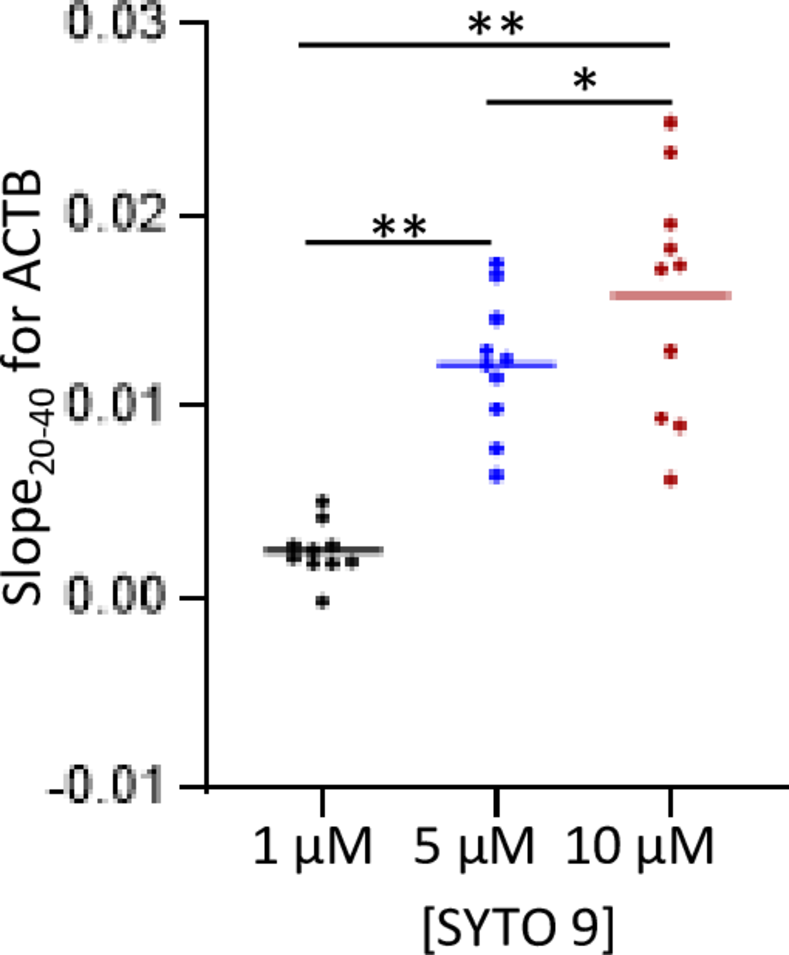

Supplement: S6 Fig — RT-LAMP for ACTB was performed with 10 clinical NP samples with the indicated SYTO 9 concentrations. Bars represented the mean slope20-40. A paired t-test was used to assess differences in the means. *, P < 0.05; **, P < 0.01. (TIF) [file pone.0268340.s006.tif]
